# The Origins of Novel Protein Interactions during Animal Opsin Evolution

**DOI:** 10.1371/journal.pone.0001054

**Published:** 2007-10-17

**Authors:** David C. Plachetzki, Bernard M. Degnan, Todd H. Oakley

**Affiliations:** 1 Ecology, Evolution and Marine Biology, University of California at Santa Barbara, Santa Barbara, California, United States of America; 2 School of Integrative Biology, University of Queensland, Brisbane, Queensland, Australia; Utrecht University, Netherlands

## Abstract

**Background:**

Biologists are gaining an increased understanding of the genetic bases of phenotypic change during evolution. Nevertheless, the origins of phenotypes mediated by novel protein-protein interactions remain largely undocumented.

**Methodology/Principle Findings:**

Here we analyze the evolution of opsin visual pigment proteins from the genomes of early branching animals, including a new class of opsins from Cnidaria. We combine these data with existing knowledge of the molecular basis of opsin function in a rigorous phylogenetic framework. We identify adaptive amino acid substitutions in duplicated opsin genes that correlate with a diversification of physiological pathways mediated by different protein-protein interactions.

**Conclusions/Significance:**

This study documents how gene duplication events early in the history of animals followed by adaptive structural mutations increased organismal complexity by adding novel protein-protein interactions that underlie different physiological pathways. These pathways are central to vision and other photo-reactive phenotypes in most extant animals. Similar evolutionary processes may have been at work in generating other metazoan sensory systems and other physiological processes mediated by signal transduction.

## Introduction

Documenting the specific genetic changes driving phenotypic evolution is a fundamental goal of current biology. Genetic changes are known to modify phenotype during evolution by altering the interactions between a protein and its ecological or biochemical environment [1]–[4], by modulating existing protein-protein interactions [5], or by changing protein-DNA interactions through regulatory mutations [6]–[9]. However, the specific genetic changes that give rise to the evolutionary origins of novel protein-protein interactions have rarely been documented in detail [10], but see [11].

Animal phototransduction pathways offer great opportunity for elucidating the genetic basis for evolutionary novelty for a number of reasons. First, a diversity of presumably ancient phototransduction pathways exists in animals [12]. Second, the composition of these cascades has been the subject of numerous functional biochemical studies. This is especially important because experimental demonstration that specific mutations cause phenotypic changes is often the most difficult aspect of a full documentation of the causal genetic changes driving phenotypic evolution [13]. Third, the proteins of animal phototransduction are amenable to phylogenetic study.

Fundamental to animal phototransduction pathways are the opsin visual pigment proteins, which bind to light reactive chromophores. As members of the G protein-coupled-receptor (GPCR) family [6], the various clades of animal opsins activate alternative G-proteins, resulting in three major phototransduction networks: ciliary, rhabdomeric, and G_o_–coupled [14]–[16]. Ciliary opsins initiate signaling through binding of a G_i/t_ α subunit of the G-protein [15], rhabdomeric opsins utilize a G_q_ α subunit in signaling [17] and class-specific G_o_ α has been identified in the G_o_-coupled opsin signaling pathway [18]–[20]. Another class of opsins, including Retinal G-protein-coupled Receptor (RGR) and retinochromes, probably do not signal through any G-protein. Instead they are involved in the re-activation of the light reactive chromophore.

Here, we couple specific functional knowledge about opsin's role in signal transduction with new phylogenetic analyses. These analyses elucidate specific genetic changes that were likely involved in the origins of the different animal phototransduction networks, which mediate various light responses of animals. Our phylogenetically based analyses indicate a significant correlation between opsins' G-protein binding phenotypes and amino acid positions in the fourth cytoplasmic loop of opsin, especially positions homologous to 310 and 312 of bovine rhodopsin. Previous biochemical analyses demonstrate that these same amino acid positions are involved in G-protein binding function. Our additional analyses indicate that G-protein binding phenotypes likely diversified at the time of opsin gene duplication events before the origin of bilaterians, and the specific amino acid changes involved retain a pattern consistent with purifying selection.

## Results

### Multiple opsin genes are present in cnidarians but absent in a demosponge

**Figure 1:**
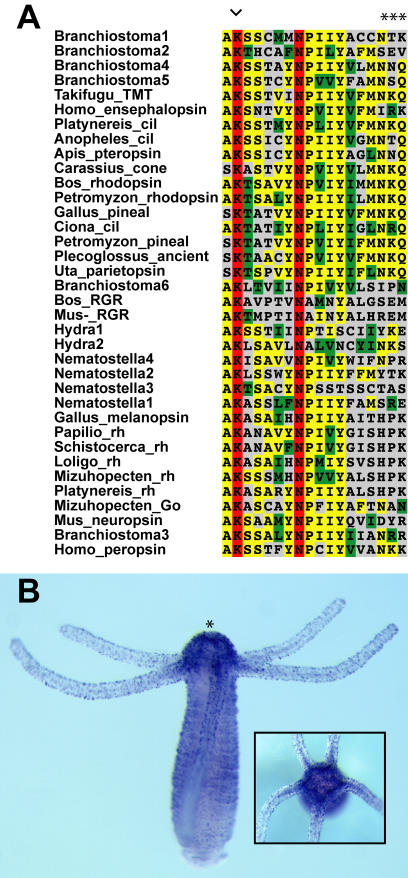
Sequence motifs and expression of cnidarian opsin in the nerve net of *Hydra magnipallata*. (A) Sequence alignment of 4^th^ cytoplasmic loop region of animal opsins used in this study indicating the Lys 296 chromophore binding site (arrowhead) and the G protein-binding tripeptide (asterisks). (B) In situ hybridization with Hm2 cnidops probe. Asterisk denotes the hypostome. Opsin is expressed most strongly in a ring of sensory neurons that surround the mouth. Inset, oral view.

Screens for opsin genes in the genome trace data from the cnidarians *Hydra magnipapillata* and *Nematostella vectensis* produced multiple unique opsins which all lack introns. Six of these cnidarian opsins were found in public Expressed Sequence Tag (EST) databases (Table S1). Consistent with opsin status, Hydra2 is expressed in the nerve net of *Hydra* based on in situ hybridization and includes sequence motif hallmarks of opsin (figure 1).

While multiple opsins were found in the cnidarian genomes, our screens for opsins in trace data from the poriferan *Amphimedon queenslandica* did not produce any putative opsins. These screens did produce several non-opsin, rhodopsin-class GPCR genes from *Amphimedon* (data not shown). We were also unable to obtain opsin sequences from the trace genome data of the placozoan *Trichoplax* or the choanoflagellate *Monosiga*. Animal (type II) opsins are also unknown from numerous fungal genomes [21].

### A new class of cnidarian opsins helps resolve phylogenetic relationships

In our discussion of unrooted phylogenies, we refrain from using the common terms “sister group” and “clade” in favor of the terms “adjacent group” and “clan” as the former terms imply a rooting hypothesis *a priori* [22].

**Figure 2:**
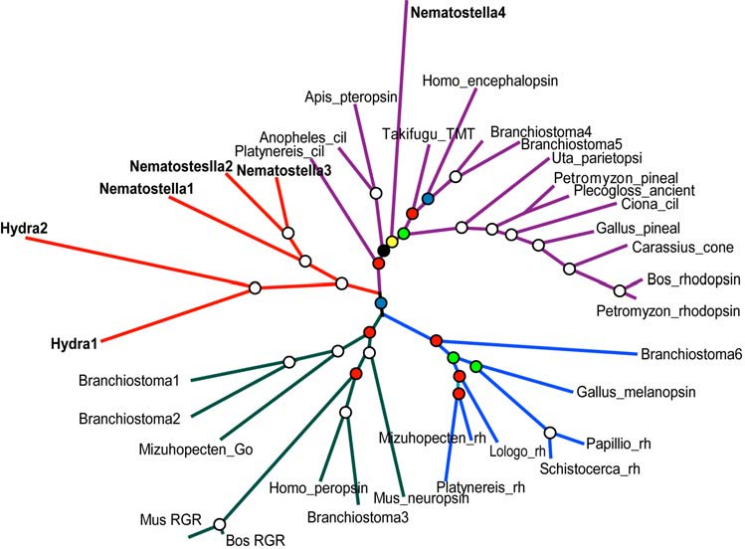
Unrooted metazoan-wide phylogeny of opsins, new cnidarian genes in bold, branches proportional to substitutions per site. Circles at nodes indicate Bayesian posterior probabilities (White = 1.0, Red>0.90, Blue>0.80, Green>0.70, Yellow>0.60, Black>0.50). cil = ciliary, rh = rhabdomeric.

Our phylogenetic analyses reveal a new clan of opsins known only in cnidarians, which we have named “cnidops”. Together, metazoan opsins form two major clans in unrooted analyses. One clan unites rhabdomeric and RGR/G_o_–coupled opsins in an adjacent group and the second consists of the newly identified cnidops family plus the ciliary opsins, which we here confirm by phylogenetic analysis to include a non-bilaterian representative (Nematostella4; figure 2).

**Figure 3:**
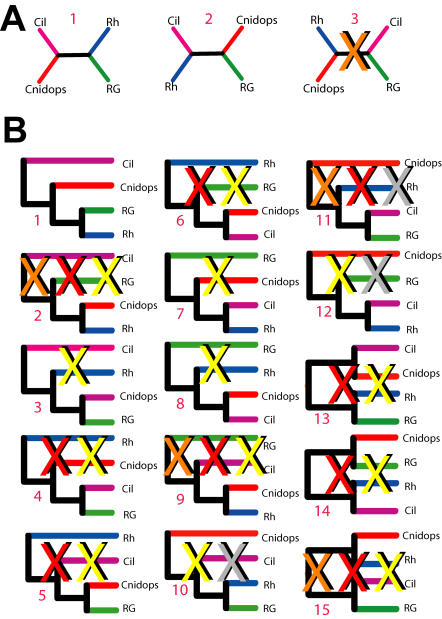
Summary of analyses to determine and root opsin phylogeny. Illustrated is each possible unrooted and rooted hypothesis, assuming monophyly of four major opsin clades. Trees in (A) 1–3 correspond to possible unrooted topologyies and those in (B) 1–15 represent all possible rooted trees, see Table 1. Orange X indicates that tree had significantly lower likelihood in opsin-only dataset. Red X indicates tree had significantly lower likelihood in opsin+outgroup dataset. Yellow X indicates that tree implies additional gene duplication/loss events compared to minimum (tree 1 is minimum with 2 duplications 0 losses implied). Grey X indicates tree with cnidops as earliest branching opsin group–a result inferred in parametric bootstrap analyses, which incorrectly grouped cnidops+outgroup opsins because of long branch-attraction (see supplementary methods). Cil = ciliary; Rh = rhabdomeric; RG = Go/RGR.

**Figure 4:**
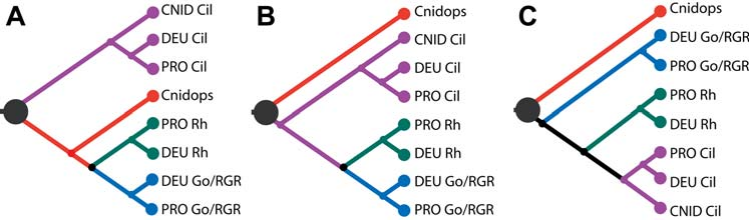
Three hypotheses for metazoan opsin relationships. These are statistically indistinguishable when considering only the likelihood of observed amino acid sequences. (A) Our preferred phylogenetic hypothesis where ciliary opsins are an outgroup to other opsin clades and cnidops is the sister to the rhabdomeric+G_o_ clade. This hypothesis minimizes the number of gene duplication and loss events required to explain the evolutionary history of metazoan opsins and is consistent with morphological data [33]. (B and C) Phylogenetic hypotheses in which cnidops represents the outgroup to other opsins. Given our finding of a ciliary opsin in *Nematostella*, these hypotheses require the additional loss of ciliary opsin in the *Hydra* lineage and an additional loss of cnidops in bilaterian animals.

Without non-opsin outgroups, our analyses of metazoan opsin phylogeny yielded well supported topologies. In order to root our tree, we used a combination of likelihood comparisons, reconciled tree analyses and parametric bootstrapping (summarized in figure 3 and described in Methods). The majority of available evidence indicates that opsin phylogeny is best rooted between the ciliary and non-ciliary clades (figure 4A).

**Table 1:** Likelihood comparisons for alternative hypotheses of opsin evolution

Tree	-ln[*L*]	Δ-ln[*L*]	+-SE −ln[*L*]	AU	pKH	pSH	pRELL	Reconciled Trees	
								Dup	Loss
A1	−15637.857	0.000	0.000	0.660	−1.000	1.000	0.581	-	-
A2	−15642.642	−1.842	7.349	0.420	0.401	0.467	0.395	-	-
A3	−15642.642	−4.785	4.032	0.076	0.118	0.301	0.024	-	-
B1	−16190.354	−0.553	1.696	0.518	0.372	0.854	0.131	0	0
B2	−16195.124	−5.324	4.625	0.038	0.125	0.345	0.002	0	1^1^, ^2^, ^3^
B3	−16192.821	−3.020	5.982	0.302	0.307	0.600	0.065	0	1^1^, ^2^, ^3^
B4	−16196.016	−6.215	4.921	0.045	0.103	0.274	0.001	0	1^1^, ^3^
B5	−16193.309	−3.508	6.185	0.150	0.285	0.552	0.013	3	2^1^, ^3^
B6	−16193.576	−3.775	3.272	0.070	0.124	0.540	0.003	0	1^1^
B7	−16192.200	−2.399	6.672	0.302	0.360	0.657	0.057	0	3^1^, ^2^, ^3^
B8	−16192.081	−2.280	4.176	0.352	0.293	0.708	0.084	1	3^1^, ^2^, ^3^
B9	−16194.972	−5.172	5.481	0.048	0.173	0.364	0.002	2	1^1^
B10	−16189.801	0.000	0.000	0.643	1.000	1.000	0.275	1	2^2^
B11	−16193.990	−4.189	3.832	0.081	0.137	0.489	0.008	0	1^2^
B12	−16190.299	−0.498	5.686	0.621	0.465	0.857	0.247	1	3^1^, ^2^, ^3^
B13	−16190.591	−0.791	1.387	0.311	0.284	0.837	0.032	0	1^2^
B14	−16191.817	−2.016	6.722	0.464	0.382	0.709	0.078	0	3^1^, ^2^, ^3^
B15	−16196.016	−6.215	4.921	0.045	0.103	0.274	0.001	0	1^2^

Likelihood comparison tests for trees in figure 3. Results were calculated under the WAG+I+G model. *L,* likelihood; AU, Approximately Unbiased Test [66]; KH, Kishino-Hasegawa test [67]; SH, Shimodaria-Hasegawa test [55]; pRELL, resampled log-likelihood bootstrap percentage [68].

1 Requires Cnidarians Lost RGR/Go Clade

2 Requires Cnidarians lost Rhabdomeric

3 Requires Bilaterians lost cnidops

### Timing of origin of major opsin clades

**Figure 5:**
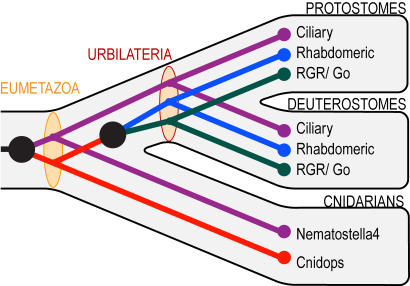
Our preferred hypothesis for opsin phylogeny (figure 4A) reconciled to a conservative view of metazoan phylogeny. Black circles indicate gene duplication events. Tan ovals indicate the opsin complement at key nodes in metazoan phylogeny. By this hypothesis, both ciliary and cnidops opsins were present in the eumetazoan ancestor of Cnidaria+Bilateria while rhabdomeric and G_o_ opsins evolved by gene duplication prior to the evolution of bilaterian animals, but not before.

Using our rooted opsin phylogeny, reconciled tree analyses (RTA) provide new information on the timing of origin of the various opsin clades (figure 5). RTA indicates that the ciliary and cnidops lineages originated by gene duplication at or before the cnidarian-bilaterian common ancestor (Eumetazoa), approximately 600 million years ago [23], [24].

Of the two ancient metazoan opsin lineages, one is represented in extant taxa by the newly described cnidops opsins of cnidaria, RGR/G_o_ and rhabdomeric opsins. Our results concur with previous minima for the origins of RGR/G_o_ and rhabdomeric opsins at the protostome-deuterostome common ancestor (Urbilateria) [14], [25] and provide a new maximum for the origin of these clades by a gene duplication event younger than the eumetazoan ancestor (figure 5). A second ancient opsin lineage survives as the ciliary opsins and includes one cnidarian opsin (Nematostella4) and those of the vertebrate rods, cones, pineal and parapineal, and of invertebrate extra-ocular cells [25], [26]. Despite finding the *Nematostella* gene, we did not find any *Hydra* genes from this ciliary opsin clade.

### The early evolution of G protein binding partners

**Figure 6:**
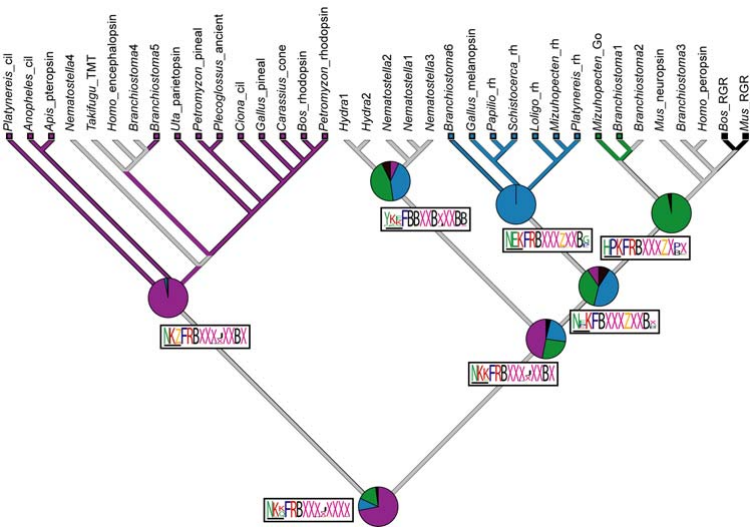
Ancestral state reconstruction of G protein-binding interactions for each metazoan opsin-mediated phototransduction cascade obtained by simulated mutational mapping [64] (see methods). For each class of opsin, the P value of the reconstructed ancestral G α interactions is represented in pie graphs. Ancestral G protein interactions in phototransduction cascades mediated by ciliary, rhabdomeric and G_o_ opsins can be significantly resolved (P>0.95) but the ancestral states of the rhabdomeric+G_o_, and cnidops clades are equivocal. ML state reconstructions shown for each node as colored branches. Red, G_i/t_; Blue, G_q_; Green, G_o_; Black, no G protein interaction (as is the case for RGR/Retinochrome opsins); Grey, equivocal reconstruction from ML. Reconstructed ancestral amino acid motifs of the 4th cytoplasmic loop region of opsin are shown along branches in logos. Maximum vertical height scales to P  = 1.0. We obtained clear reconstructed states for most of the residues in a conserved tripeptide motif (residues 310, 311 and 312, horizontal bar) for the ciliary, rhabdomeric and G_o_ /RGR nodes. For the most part, the remainder of the residues in can be unequivocally reconstructed to the level of Dayhoff classes. B = HRK, X = LIVM, J = GATSP, Z = DENQ.

**Figure 7:**
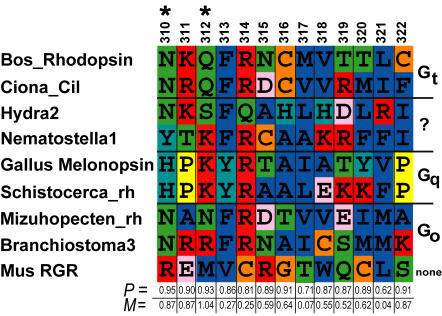
Summary of co-variation analyses and tests for selection. Previously described G protein binding states, together with amino acid sequences of the 4^th^ cytoplasmic loop regions from our opsin dataset, were used to test the hypothesis that the two character sets co-vary across our opsin phylogeny. Co-variation was assessed using mutual information content (MIC). Shown here is a representation of these results. Predictive P-values (Bottom axis) based on MIC for co-variation between each residue in the 4^th^ cytoplasimic loop and their respective G protein interactions (Right axis) are shown. These analyses were conducted using Bayesian mutational simulation mapping in SIMMAP [64]. We also tested possibility that these residues had evolved under a selective regime using the criterion of site specific rate heterogeneity as implemented in DIVERGE2 [29]. The two highest scoring residues from co-variation analyses (310 and 312) also retain the signature of selection (asterisk). See table S1 for citations. *P* = predictive P-value, *M* = the M statistic given by MIC.

Our phylogeny provides a framework for understanding key transitions in the evolution of animal phototransduction pathways. Opsins interact with their corresponding G proteins, in part through binding at conserved sequence motifs located in opsin's fourth cytoplasmic loop [27]. We obtained clear reconstructed ancestral states for most of the residues in a conserved tri-peptide motif for the ciliary, rhabdomeric and RGR/G_o_ nodes. For the most part, the remainder of the residues in the fourth cytoplasmic loop can be unequivocally reconstructed to the level of Dayhoff classes (i.e., C/HRK/FYW/DENQ/LIVM/GATSP) [28] (figure 6). In addition, many of the residues of this region significantly co-vary with G protein α subunit interaction on our phylogeny. Two of the highest scoring residues in the co-variation analysis, residues 310 and 312 corresponding to positions 1 and 3 of the G protein-binding tripeptide motif [27], possessed a signature of selection using algorithms in DIVERGE2 [29] (figure 7).

We also explored the character history of G protein binding interactions by reconstructing their ancestral states across our phylogeny (figure 6). While we cannot conclusively resolve the G protein interaction in the ancestor of rhabdomeric+G_o_ opsins or the ancestor of ciliary+rhabdomeric+G_o _clades, our reconstructed ancestral G protein interaction states for ciliary, rhabdomeric and G_o_ are highly significant (P>0.95 for each) and are congruent with previous empirical biochemical studies [17], [19], [30] (figure 6).

## Discussion

Our findings provide several new insights into the evolution of animal phototransduction cascades. First, we identify a new class of opsins that appears to be unique to cnidarians. Second, we find no evidence of any opsins in genomes of a demosponge, a parazoan, a choanoflagellate or fungi. We conclude that opsin-mediated phototransduction cascades originated early in eumetazoan evolution, after this lineage diverged from the demosponge lineage. The rhabdomeric and G_o_-coupled cascades appear to be bilaterian innovations. Third, the origins of novel phototransduction cascades, as defined by the evolution of alternative opsin-G protein interactions, co-occur on our phylogeny with the gene duplications that have given rise to the major opsin classes. Fourth, the specific mutational events that have lead to the functional diversification of the major opsin lineages are suggested by co-variation analyses and tests for selection. These results demonstrate how gene duplications, together with structural mutations that lead to novel protein-protein interactions, can contribute to the evolution of novel physiological traits. Each of these points is discussed further below.

### The timing and relationships of animal opsins

The inter-relationships of animal opsins reported here are based on thorough phylogenetic analyses that included standard phylogenetic tree estimation, comprehensive likelihood comparisons, RTA, and parametric bootstrapping (figure 3). Taken together, the majority of evidence favors the hypothesis depicted in figure 4A, although the trees in figures 4B and C remain viable alternative hypotheses. However, because these latter hypotheses require additional gene duplication and loss events to explain the rooted topology, and because parametric bootstrapping simulations indicate that long branch lengths can contribute to the erroneous resolution of specifically these topologies, we favor the former hypothesis (figure 4A). Given present sampling and the assumption of monophyly of major opsin clades, any other phylogenetic hypotheses are significantly rejected by the data. Even if figure 4A does not represent the true history of opsin, the two viable alternative hypotheses (figures 4B and C) do not drastically alter our interpretation of the timing of origins of animal opsin in RTA analysis. Irrespective of which hypothesis we consider, animal opsins originate at the same node (Eumetazoa). If we assume the hypothesis depicted in figure 4B our conclusion that RGR/G_o_-coupled and rhabdomeric opsins are bilaterian synapomorphies also remains intact, but requires an additional loss of cnidops prior to Bilateria. The latter is also true of the topology shown in figure 4C.

A maximum date for the origin of animal opsins (when a GPCR first bound a light reactive chromophore) is equivocal because we did not obtain any opsins in the genome sequence of an earlier branching animal, the demosponge *Amphimedon queenslandica*, or from any non-animal genomes. While the larvae of *Amphimedon* are capable of phototaxis, studies of spectral sensitivity have suggested the activities of a flavin-based photoreceptor in this behavior [31], as opposed to retinal as used by opsins. Further, we uncovered no animal (type II) opsins from bioinformatic screens of the placozoan *Trichoplax* or the choanoflagelate *Monosiga* genomes and no true homolog of type II opsins have been described outside of Metazoa [32]. While we cannot rule out an earlier opsin origin coupled with loss in lineages leading to the species with genome sequences, we strongly favor the hypothesis that animal opsins were present in the eumetazoan ancestor of cnidarians and bilaterians, but not at earlier nodes in eukaryote phylogeny. Since porifera, *Monosiga*, and fungi seem to lack opsins, yet possess rhodopsin-class GPCR proteins without the Lysine residue to bind a light reactive chromophore, one possibility is that a rhodopsin-class GPCR gained the ability to bind a chromophore by substitution to a Lysine residue during animal evolution.

Our results contrast with previous studies on opsin relationships that placed ciliary and G_o_-coupled opsins as sister groups [14], or adjacent groups [15], and those that placed ciliary and rhabdomeric opsins as sister groups [12]. However, in lacking data from non-bilaterian animals, these previous works were able to include only three of the four animal opsin lineages discussed here. The new clade of opsins from the Cnidarian, “cnidops”, has aided in the resolution of the overall opsin phylogeny, especially because non-opsin outgroups seem to destabilize ingroup topology.

The presence of an opsin from the cnidarian *Nematostella* that in our analyses groups within the ciliary clade has been foreshadowed by previous morphological descriptions of ciliary photoreceptors [33], [34], but no previous opsin data from the group have confirmed this hypothesis. Our ability to find only cnidops and not ciliary opsins in *Hydra* probably indicates that ciliary clade opsins were lost during this species' evolutionary history.

### The genetic basis for opsin-G protein interactions

Multiple lines of evidence support the hypothesis that amino acid substitutions in the 4th cytoplasmic loop of duplicated opsins were involved in the origins of novel opsin-G protein interactions. First, specific amino acid states significantly co-vary with G-protein activation phenotypes during opsin phylogeny (figure 7). Because we used a Bayesian approach that estimated the posterior probability of all possible trees, the estimated correlation accounts for uncertainty in opsin phylogeny. The highest p-values in these correlation analyses correspond to residues formerly demonstrated by site-directed mutagenesis to mediate opsin-G protein interaction in ciliary opsins (i.e., residues 310 and 312 [27]).

These amino acid changes retain the signature of selection, as given by site-specific shifts in evolutionary rates, a statistical test that uncovers significant conservation within gene clades compared to diversification between gene clades [29] (figure 7). Although this pattern cannot be used to determine if the mutations were originally fixed by natural selection, conservation after an ancient diversification indicates a long period of stasis, consistent with purifying selection on adaptive, alternative amino acid states. The combination of correlation, functional tests, and a pattern consistent with purifying selection strongly suggests that substitutions in residues homologous to bovine opsin 310 and 312 contributed to the origin of novel phototransduction pathways with alternative G-protein binding partners.

The pattern of early divergence followed by conservation is also evident in our estimates of ancestral opsin-G protein binding phenotypes. Using available data, we were able to unequivocally resolve the ancestral G-protein usage phenotypes for the ciliary, rhabdomeric and RGR/G_o_-coupled opsin clades (figure 6). That we can resolve the G protein binding specificities at the base of each of these clades, but not at deeper nodes, suggests an early diversification of G protein binding specificities, coupled with relative stasis of protein interactions once the major opsin clades were established.

Because we find that opsins were present in extant eumetazoans but not in animals representing earlier branching lineages (i.e. demosponges), the evolution of novel phototransduction cascades in animals probably proceeded by evolving specialized protein-protein interactions between new opsin paralogs and existing G protein signaling intermediaries, an evolutionary process previously described as molecular exploitation [4]. Each of the G α subunit paralogs known to interact with opsins in bilaterians has also been described from plants, fungi, protists, cnidarians and sponges indicating a long pre-animal history for the different G-proteins [35], [36].

While our inclusion of new data from the Cnidaria has greatly enhanced our ability to reconstruct opsin phylogeny, functional or phenotypic data from the newly reported cnidops clade do not exist. Further studies on the biochemistry of signaling by cnidops are required if we are to better understand the history of opsin-G protein interactions in this important family of opsins. Despite this missing data, P-values for our ancestral state reconstructions for the remaining opsin classes suggest that our results are robust.

### Implications for early animal sensory systems

Our finding of an adaptive, early partitioning of opsin-mediated signaling into discrete functional classes in the course of their evolution provides insight into the organismal properties of the ancestor of cnidarian and bilaterian animals. The activities of ciliary, rhabdomeric and G_o_-coupled opsins are known to exist in close proximity [37], if not the same cell [30], in numerous taxa. The early, adaptive evolution of the different photosensory pathways suggested by our analyses may be an indication of the functional necessity to transduse these signaling cascades through non-overlapping channels in the eumetazoan ancestor, an animal that by our analysis possessed ancestral ciliary and ancestral cnidops+rhabdomeric+RGR/G_o_-clade opsins. Such early functional requirements could have included the need to delineate light information used for vision from that used to entrain the circadian clock cycle [38] or to differentiate between light of differing wavelengths, two core processes accomplished by specific cell types in bilaterian animals [12].

The proposed relationship between gene duplication and biological complexity has been invoked and re-invoked at various points throughout the past century of biological investigation [39], and was most famously articulated by Ohno [40]. Despite the great level of interest in models for the evolution of complexity based on gene duplication, data demonstrating how gene duplication can be implicated in the origins of novel protein-protein interactions are not common. Here we begin to fill this gap and show that a group of familiar signaling pathways, animal phototransduction cascades, provides a useful model for the study of the origins of novel phenotypes.

## Materials and Methods

### Data mining

Publicly available trace genome sequence data from *Hydra magnipapillata*, *Nematostella vectensis* and *Amphimedon queenslandica* (http://www.ncbi.nlm.nih.gov/Traces) were subjected to tblastn [41] searches using a wide range of homologous opsin proteins from bilaterian animals (Table S1). We implemented an in-house assembly pipeline, where BLAST hits were extended to their largest possible contigs under stringent parameters, using only clean trace data as determined using PHRAP [42] and an 85% identity cut off for joining trace fragments. Genes were predicted from genomic contigs using GenomeScan [43]. A combination of BLAST searches, phylogenetic analyses and sequence motif analyses were used to establish the authenticity of opsin sequences recovered from genome analyses. In particular, the residue homologous to the bovine lysine 296, which binds a light reactive chromophore [44], has been used to validate opsin identity (figure 1). We corroborated the existence of our predicted proteins by using EST databases at either NCBI (http://www.ncbi.nlm.nih.gov/), for *Hydra*, or the Joint Genome Institute, for *Nematostella* (http://www.jgi.doe.gov/). Homologous proteins and outgroup data from bilaterian animals used in phylogenetic analyses were also obtained from NCBI (Table S1).

### Animal culture and in situ hybridization

*Hydra magnipapillata* (UC Irvine strain) were reared in the laboratory under standard conditions at 18°C. The expression of one opsin, HM2, was investigated in *Hydra magnipapillata* using in situ hybridization following Grens et al. [45]. The following modifications were made: A pCRII vector (Invitrogen) containing HM2 opsin cDNA (∼750 bp) was used as template for probe synthesis. DIG-labeled probes were synthesized using T3 and T7 polymerase (Roche). Both sense and anti-sense probes (0.3 µg/ml) were hybridized at 55°C for 48 to 60 hours. Sense probes produced no detectable signal.

### Phylogenetic approaches

For phylogenetic analyses, only the 7-transmembrane region including intervening inter- and extra-cellular domains was included (330 amino acids), as it was difficult to ascertain homology of N- and C- termini due to sequence length variation and lack of conservation across genes. Taxon/gene selection was done in a manner that enhanced both the taxonomic representation across the Metazoa and the inclusion of well-studied subfamilies (i.e., arthropod Rh opsins and vertebrate visual opsins), but also allowed for thorough computational analyses. Protein sequences were aligned using T-COFFEE under default parameters [46] and alignment manipulations were done using Seaview [47]. Phylogenetic analyses were conducted using unweighted Maximum Parsimony (MP) implemented in PAUP* 4.0b10 [48], Maximum Likelihood (ML) implemented in PHYML v2.4.4 [49], and Bayesian Markov Chain Monte Carlo (BMCMC) implemented in MrBayes 3.1 [50]. Support for internal nodes was assessed with 1000 bootstrap replicates for MP and ML, and posterior probability for BMCMC analyses. ML and BMCMC approaches assumed best-fit models of protein evolution, as determined using ProtTest [51]. For ML tree calculations, both the proportion of invariant sites and the α parameter of the gamma distribution were estimated and both tree topology and branch lengths were optimized in PHYML. Bayesian analyses were conducted for 20 million generations using default priors and heating parameters for each of four chains. For the monitoring of progress in BMCMC runs, we assumed convergence of the Markov Chains when standard deviation of split frequencies (SDSF) fell below 0.01, indicating that the two independent runs resulted in similar phylogenies [50]. Following the BMCMC runs, burnin was assessed using Tracer [52] and by the SDSF of the two independent BMCMC runs. For un-rooted analyses, the initial 11 million generations with an SDSF of 0.0015 or greater were discarded. For rooted analyses the first 12 million generations with an SDSF of 0.0024 or greater were removed prior to consensus tree calculation.

The evolutionary relationships between members of the larger GPCR class of proteins remain poorly understood. Because of this, our outgroup selection in rooted analyses was empirical. We selected the shortest-branched sequences from a pool of rhodopsin class GPCRs [32] from a variety of taxa (Table S1) after non-bootstrapped ML and shorter (i.e., 3-5 million steps) BMCMC analyses.

### Tests of phylogenetic hypotheses

In order to address the lack of resolution among the major opsin clades encountered in phylogenetic analyses that included outgroups (figure S2), we tested the significance of a wide range of competing phylogenetic hypotheses for metazoan opsin. First, we assumed monophyly of each of the four major opsin clades (cnidarian opsin, ciliary, RGR/G_o_ and rhabdomeric). This assumption is supported by the results of unrooted MP, ML and BMCMC analyses (figure S2) and by previous studies on opsin phylogeny [12], [15]. Our topology contains four opsin clades that can be rearranged in 15 possible rooted binary trees. Second, we calculated the likelihood for each possible tree using the “resolve multifurcations” function in TREEFINDER [53]. Finally, we used CODEML, as included in PAML [54], to assess the significance of each of these trees. These results were further analyzed using CONSEL [55]. A similar approach was used to assess alternative topologies in unrooted trees where having four major clades involves three possible unrooted topologies (figure 4).

### Parametric bootstrapping

Given the ambiguous results obtained when including non-opsin outgroups, we suspected that long branch-attraction (LBA) artifacts [56], where the cnidops sequences were pulled to the base of the tree by long branch outgroups, were confounding our analyses. We tested this hypothesis by using Huelsenbeck's [57] method of parametric bootstrapping. Here, a tree including a non-opsin outgroup was constrained to reflect the best topology consistent with Tree 1B in figure 3. Assuming this tree and the best-fit model of opsin molecular evolution from prot-test analysis [51] , we simulated 100 data sets using SeqGen [58]. Each of these simulated data sets was subjected to ML and MP analyses. Since the data were simulated, we expected to recover the assumed phylogeny unless the topology/model combination is sensitive to artifacts like long branch-attraction [57]. For ML analyses we found that the simulated cnidarian genes are incorrectly attracted to the base of the tree in 45% of replicates, whereas the correct, simulated topology was recovered in only 35% of the replicates. In MP analyses, the cnidarian opsin genes were incorrectly attracted to the outgroup in 49% of the replicates, while the simulated phylogeny was again recovered in only 35% of cases. These simulations show that analyses including opsin outgroups are sensitive to artifacts that induce erroneous topology estimates. Our results are similar to those reported for analyses of strepsipteran relationships, a well-known case of LBA [57].

### Reconciled tree analysis

The number of gene duplication and loss events that are implied by a given rooting hypothesis can be used to assess the chances that a given hypothesis is tenable [59]. We compared the number of duplications and losses implied by each possible root position of our unrooted phylogeny using NOTUNG [60], [61]. For these analyses we assumed a conservative species-level phylogeny for the major taxa included in our analyses: Hydrozoa, Anthozoa, Cephalochordata, Urochordata, Vertebrata, Annelida, Mollusca, and Arthropoda [62] .

### Character mapping and tests of co-variance

We scored G-protein interaction phenotypes for the opsins in our phylogenetic analysis as a discrete state character. Although these phenotypes are unknown for many opsins, especially those from non-model organisms, our analysis includes interaction data for 48% of the opsin sequences represented on our phylogeny (figures 6, S1).

Ancestral state reconstructions and tests for correlated character evolution were conducted using Bayesian mutational mapping [63] as implemented in SIMMAP [64]. Reconstructions were integrated over 18,000 trees that were sampled from our unrooted BMCMC analyses. Ancestral state reconstructions were conducted with 10 realizations sampled from the prior and 10 realizations sampled for each tree. We set an equal prior on the bias parameter but did not abstract a prior for the rate parameter, instead using branch lengths from BMCMC trees as relative rates. As there is no *a priori* reason to assume different prior parameters, we chose this model, as it is the least parameterized model for morphological evolution possible in SIMMAP [64]. We also tested a range of alternative prior settings and obtained similar P-values in these analyses (results not shown). Tests for selection were conducted in DIVERGE2 [29] using our amino acid alignment (see supplemental materials), rooted phylogeny and the structure of bovine opsin previously calculated by x-ray crystallography [65]. Our estimate for site specific rate shift (functional divergence) between ciliary and cnidops+RGR/G_o_+Rhabdomeric was significantly larger than 0: θ_1_ = 5.82±0.05. Site-specific posterior probabilities for residues 310 and 312 were 0.45 and 0.37 respectively.

## Supporting Information

Table S1 Sequences used in opsin phylogenetic analyses
(0.11 MB DOC)

Figure S1 Phylogenetic analyses of opsins without an outgroup A. ML analyses were conducted assuming the WAG +I+G+F model. B. Unweighted MP. Node numbers represent bootstrap proportions out of 1000 replicates.
(3.31 MB TIF)

Figure S2 Results of analyses when including non-opsin outgroup. A. BMCMC, B. MP, and C. ML. BMCMC and ML analyses were conducted under the WAG+I+G+F model. Similar analyses with other outgroups produced qualitatively similar results (not shown)
(6.19 MB DOC)

## References

[1] Yokoyama S (2000). Molecular evolution of vertebrate visual pigments.. Prog Retin Eye Res.

[2] Spaethe J, Briscoe AD (2005). Molecular characterization and expression of the UV opsin in bumblebees: three ommatidial subtypes in the retina and a new photoreceptor organ in the lamina.. J Exp Biol.

[3] Zhang J, Zhang YP, Rosenberg HF (2002). Adaptive evolution of a duplicated pancreatic ribonuclease gene in a leaf-eating monkey.. Nat Genet.

[4] Bridgham JT, Carroll SM, Thornton JW (2006). Evolution of hormone-receptor complexity by molecular exploitation.. Science.

[5] Hoekstra HE, Hirschmann RJ, Bundey RA, Insel PA, Crossland JP (2006). A single amino acid mutation contributes to adaptive beach mouse color pattern.. Science.

[6] Shapiro MD, Marks ME, Peichel CL, Blackman BK, Nereng KS (2004). Genetic and developmental basis of evolutionary pelvic reduction in threespine sticklebacks.. Nature.

[7] Bachner-Melman R, Dina C, Zohar AH, Constantini N, Lerer E (2005). AVPR1a and SLC6A4 gene polymorphisms are associated with creative dance performance.. PLoS Genet.

[8] Rockman MV, Hahn MW, Soranzo N, Zimprich F, Goldstein DB (2005). Ancient and recent positive selection transformed opioid cis-regulation in humans.. PLoS Biol.

[9] Tishkoff SA, Reed FA, Ranciaro A, Voight BF, Babbitt CC (2007). Convergent adaptation of human lactase persistence in Africa and Europe.. Nat Genet.

[10] Lynch M (2007). The evolution of genetic networks by non-adaptive processes.. Nat Rev Genet.

[11] Lynch VJ, Roth JJ, Takahashi K, Dunn CW, Nonaka DF (2004). Adaptive evolution of HoxA-11 and HoxA-13 at the origin of the uterus in mammals.. Proc Biol Sci.

[12] Arendt D (2003). Evolution of eyes and photoreceptor cell types.. Int J Dev Biol.

[13] Hoekstra HE, Coyne JA (2007). The locus of evolution: evo devo and the genetics of adaptation.. Evolution Int J Org Evolution.

[14] Arendt D, Wittbrodt J (2001). Reconstructing the eyes of Urbilateria.. Transactions of the Royal Society of London B.

[15] Terakita A (2005). The opsins.. Genome Biol.

[16] Raible F, Tessmar-Raible K, Arboleda E, Kaller T, Bork P (2006). Opsins and clusters of sensory G-protein-coupled receptors in the sea urchin genome.. Dev Biol.

[17] Hardie RC, Raghu P (2001). Visual transduction in Drosophila.. Nature.

[18] Kojima D, Terakita A, Ishikawa T, Tsukahara Y, Maeda A (1997). A novel Go-mediated phototransduction cascade in scallop visual cells.. J Biol Chem.

[19] Koyanagi M, Terakita A, Kubokawa K, Shichida Y (2002). Amphioxus homologs of Go-coupled rhodopsin and peropsin having 11-cis- and all-trans-retinals as their chromophores.. FEBS Lett.

[20] Koyanagi M, Kubokawa K, Tsukamoto H, Shichida Y, Terakita A (2005). Cephalochordate melanopsin: evolutionary linkage between invertebrate visual cells and vertebrate photosensitive retinal ganglion cells.. Curr Biol.

[21] Dunlap JC, Loros JJ, Briggs WR, Spudich JL (2005). Neurospora Photoreceptors.. Handbook of Photosensory Receptors.

[22] Wilkinson M, McInerney JO, Hirt RP, Foster PG, Embley TM (2007). Of clades and clans: terms for phylogenetic relationships in unrooted trees.. Trends in Ecology and Evolution.

[23] Peterson KJ, Lyons JB, Nowak KS, Takacs CM, Wargo MJ (2004). Estimating metazoan divergence times with a molecular clock.. Proceedings of the National Academy of Sciences of the United States of America.

[24] Peterson KJ, Butterfield NJ (2005). Origin of the Eumetazoa: testing ecological predictions of molecular clocks against the Proterozoic fossil record.. Proc Natl Acad Sci U S A.

[25] Arendt D, Tessmar-Raible K, Snyman H, Dorresteijn AW, Wittbrodt J (2004). Ciliary photoreceptors with a vertebrate-type opsin in an invertebrate brain.. Science.

[26] Velarde RA, Sauer CD, Walden KK, Fahrbach SE, Robertson HM (2005). Pteropsin: a vertebrate-like non-visual opsin expressed in the honey bee brain.. Insect Biochem Mol Biol.

[27] Marin EP, Krishna AG, Zvyaga TA, Isele J, Siebert F (2000). The amino terminus of the fourth cytoplasmic loop of rhodopsin modulates rhodopsin-transducin interaction.. J Biol Chem.

[28] Hrdy I, Hirt RP, Dolezal P, Bardonova L, Foster PG (2004). Trichomonas hydrogenosomes contain the NADH dehydrogenase module of mitochondrial complex I.. Nature.

[29] Zheng Y, Xu D, Gu X (2007). Functional divergence after gene duplication and sequence-structure relationship: a case study of G-protein alpha subunits.. J Exp Zoolog B Mol Dev Evol.

[30] Su CY, Luo DG, Terakita A, Shichida Y, Liao HW (2006). Parietal-eye phototransduction components and their potential evolutionary implications.. Science.

[31] Leys SP, Cronin TW, Degnan BM, Marshall JN (2002). Spectral sensitivity in a sponge larva.. J Comp Physiol A Neuroethol Sens Neural Behav Physiol.

[32] Spudich JL, Yang CS, Jung KH, Spudich EN (2000). Retinylidene proteins: structures and functions from archaea to humans.. Annu Rev Cell Dev Biol.

[33] Eakin RM, Westfall JA (1962). Fine Structure of Photoreceptors in the Hydromedusan, Polyorchis Penicillatus.. Proc Natl Acad Sci U S A.

[34] Eakin RM (1979). Evolutionary Significance of Photoreceptors: In Retrospect.. American Zoologist.

[35] Suga H, Koyanagi M, Hoshiyama D, Ono K, Iwabe N (1999). Extensive gene duplication in the early evolution of animals before the parazoan-eumetazoan split demonstrated by G proteins and protein tyrosine kinases from sponge and hydra.. J Mol Evol.

[36] O'Halloran DM, Fitzpatrick DA, McCormack GP, McInerney JO, Burnell AM (2006). The molecular phylogeny of a nematode-specific clade of heterotrimeric G-protein alpha-subunit genes.. J Mol Evol.

[37] Gomez MP, Nasi E (2000). Light transduction in invertebrate hyperpolarizing photoreceptors: possible involvement of a Go-regulated guanylate cyclase.. J Neurosci.

[38] Gehring W, Rosbash M (2003). The coevolution of blue-light photoreception and circadian rhythms.. J Mol Evol.

[39] Taylor JS, Raes J (2004). Duplication and divergence: the evolution of new genes and old ideas.. Annu Rev Genet.

[40] Ohno S (1970). Evolution by gene duplication..

[41] Altschul SF, Madden TL, Schaffer AA, Zhang J, Zhang Z (1997). Gapped BLAST and PSI-BLAST: a new generation of protein database search programs.. Nucleic Acids Research.

[42] Ewing B, Green P (1998). Base-calling of automated sequencer traces using phred. II. Error probabilities.. Genome Res.

[43] Yeh RF, Lim LP, Burge CB (2001). Computational inference of homologous gene structures in the human genome.. Genome Res.

[44] Palczewski K, Kumasaka T, Hori T, Behnke CA, Motoshima H (2000). Crystal structure of rhodopsin: A G protein-coupled receptor.. Science.

[45] Grens A, Gee L, Fisher DA, Bode HR (1996). CnNK-2, an NK-2 homeobox gene, has a role in patterning the basal end of the axis in hydra.. Dev Biol.

[46] Notredame C, Higgins DG, Heringa J (2000). T-Coffee: A novel method for fast and accurate multiple sequence alignment.. J Mol Biol.

[47] Galtier N, Gouy M, Gautier C (1996). SEAVIEW and PHYLO_WIN: two graphic tools for sequence alignment and molecular phylogeny.. Comput Appl Biosci.

[48] Swofford D (2002). PAUP*. 4.1b10 ed..

[49] Guindon S, Gascuel O (2003). A simple, fast, and accurate algorithm to estimate large phylogenies by maximum likelihood.. Syst Biol.

[50] Ronquist F, Huelsenbeck JP (2003). MrBayes 3: Bayesian phylogenetic inference under mixed models.. Bioinformatics.

[51] Abascal F, Zardoya R, Posada D (2005). ProtTest: selection of best-fit models of protein evolution.. Bioinformatics.

[52] Rambaut A, Drummond A (2003). Tracer. MCMC Trace Analysis Tool. 1.0 ed..

[53] Jobb G, von Haeseler A, Strimmer K (2004). TREEFINDER: a powerful graphical analysis environment for molecular phylogenetics.. BMC Evol Biol.

[54] Yang Z (1997). PAML: a program package for phylogenetic analysis by maximum likelihood.. Comput Appl Biosci.

[55] Shimodaira H, Hasegawa M (2001). CONSEL: for assessing the confidence of phylogenetic tree selection.. Bioinformatics.

[56] Felsenstein J (1978). Cases in which parsimony or compatibility methods will be positively misleading.. Systematic Zoology.

[57] Huelsenbeck JP (1997). Is the Felsenstein zone a fly trap?. Systematic Biology.

[58] Rambaut A, Grassly NC (1997). Seq-Gen: An application for the Monte Carlo simulation of DNA sequence evolution along phylogenetic trees.. Computer Applications in the Biosciences in press.

[59] Thornton JW, DeSalle R (2000). Gene family evolution and homology: genomics meets phylogenetics.. Annu Rev Genomics Hum Genet.

[60] Chen K, Durand D, Farach-Colton M (2000). NOTUNG: a program for dating gene duplications and optimizing gene family trees.. J Comput Biol.

[61] Durand D, Halldorsson BV, Vernot B (2006). A hybrid micro-macroevolutionary approach to gene tree reconstruction.. J Comput Biol.

[62] Telford MJ (2006). Animal phylogeny.. Curr Biol.

[63] Nielsen RH, JP (2001). Detecting positively selected amino acid sites using posterior predictive p-values. Pacific Symposium on Biocomputing, Proceedings: World Scientific..

[64] Bollback JP (2006). SIMMAP: stochastic character mapping of discrete traits on phylogenies.. BMC Bioinformatics.

[65] Li J, Edwards PC, Burghammer M, Villa C, Schertler GF (2004). Structure of bovine rhodopsin in a trigonal crystal form.. J Mol Biol.

[66] Shimodaira H (2002). An approximately unbiased test of phylogenetic tree selection.. Syst Biol.

[67] Kishino H, Hasegawa M (1989). Evaluation of the maximum likelihood estimate of the evolutionary tree topologies from DNA sequence data, and the branching order in hominoidea.. J Mol Evol.

[68] Yang Z, Rannala B (1997). Bayesian phylogenetic inference using DNA sequences: a Markov Chain Monte Carlo Method.. Mol Biol Evol.

