# Supplementary material for: The Origins of Novel Protein Interactions during Animal Opsin Evolution
Source: PLoS One. 2007 Oct 17;2(10):e1054. doi: 10.1371/journal.pone.0001054 (PMC2013938; doi:10.1371/journal.pone.0001054)
Supplement: Table S1 — Sequences used in opsin phylogenetic analyses (0.11 MB DOC) [file pone.0001054.s001.doc]

**Table S1.**  Bilaterian sequences used in opsin phylogenetic analyses.

| **Species Name** | **Gene Name** | **Accession #** | **G protein α interaction** |
| --- | --- | --- | --- |
| *Anopheles gambiae* | Anopheles_cil | XP_312503 |  |
| *Apis mellifera* | Apis_pteropsin | NP_001035057 | G_t_ [1] |
| *Bos taurus* | Bos_rhodopsin | 62460472 | G_t_ [2] |
| *Bos taurus* | Bos_RGR | NP_786969 | None [3] |
| *Branchiostoma belcheri* | Branchiostoma1 | AB050606 | G_o_ [4] |
| *Branchiostoma belcheri* | Branchiostoma2 | AB050607 |  |
| *Branchiostoma belcheri* | Branchiostoma3 | AB050610 |  |
| *Branchiostoma belcheri* | Branchiostoma4 | AB050608 |  |
| *Branchiostoma belcheri* | Branchiostoma5 | AB050609 |  |
| *Branchiostoma belcheri* | Branchiostoma6 | AB050611 | G_q_  [5] |
| *Carassius auratus* | Carassius_cone | P32310 |  |
| *Ciona intestinalis* | Ciona_cil | BAB68391 | G_t_ [6] |
| *Gallus gallus* | Gallus_pineal | P51475 | G_t_ [7] |
| *Gallus gallus* | Gallus_melanopsin | NP_001038118 | G_q_ [8] |
| *Homo sapiens* | Homo_peropsins | NP_006574 | G_t_ [3] |
| *Homo sapiens* | Homo_ensephalopsin | XP_001094239 |  |
| *Loligo forbesi* | Loligo_rh | P24603 | G_q_ [9] |
| *Mizuhopecten yessoensis* | Mizuhopecten_rh | O15973 |  |
| *Mizuhopecten yessoensis* | Mizuhopecten_Go | O15974 | G_o_ [10,11] |
| *Mus musculus* | Mus_neuropsin | NP_861418 |  |
| *Mus musculus* | Mus_RGR | AAC69836 | None [12] |
| *Papilio glaucus* | Papilio_rh | AAD29445 |  |
| *Petromyzon marinus* | Petromyzon_pineal | Q98980 | G_t_ [13] |
| *Platynereis dumerilii* | Platynereis_rh | CAC86665 | Gq [14] |
| *Platynereis dumerilii* | Platynereis_cil | AAV63834 | G_t_ [15] |
| *Plecoglossus altivelis* | Plecoglossus_ancient | AAC41240 |  |
| *Schistocerca gregaria* | Schistocerca_rh | Q94741 | G_q_ [16] |
| *Takifugu rubripes* | Takifugu_TMT | NP_001027778 |  |
| *Uta stansburiana* | Uta_parietopsin | AAZ79904 | G_t_ + G_o_ [17] |

1. Velarde RA, Sauer CD, Walden KK, Fahrbach SE, Robertson HM (2005) Pteropsin: a vertebrate-like non-visual opsin expressed in the honey bee brain. Insect Biochem Mol Biol 35: 1367-1377.

2. Marin EP, Krishna AG, Zvyaga TA, Isele J, Siebert F, et al. (2000) The amino terminus of the fourth cytoplasmic loop of rhodopsin modulates rhodopsin-transducin interaction. J Biol Chem 275: 1930-1936.

3. Chen P, Hao W, Rife L, Wang XP, Shen D, et al. (2001) A photic visual cycle of rhodopsin regeneration is dependent on Rgr. Nat Genet 28: 256-260.

4. Koyanagi M, Terakita A, Kubokawa K, Shichida Y (2002) Amphioxus homologs of Go-coupled rhodopsin and peropsin having 11-cis- and all-trans-retinals as their chromophores. FEBS Lett 531: 525-528.

5. Koyanagi M, Kubokawa K, Tsukamoto H, Shichida Y, Terakita A (2005) Cephalochordate melanopsin: evolutionary linkage between invertebrate visual cells and vertebrate photosensitive retinal ganglion cells. Curr Biol 15: 1065-1069.

6. Nakashima Y, Kusakabe T, Kusakabe R, Terakita A, Shichida Y, et al. (2003) Origin of the vertebrate visual cycle: genes encoding retinal photoisomerase and two putative visual cycle proteins are expressed in whole brain of a primitive chordate. J Comp Neurol 460: 180-190.

7. Matsushita A, Yoshikawa T, Okano T, Kasahara T, Fukada Y (2000) Colocalization of pinopsin with two types of G-protein alpha-subunits in the chicken pineal gland. Cell Tissue Res 299: 245-251.

8. Contin MA, Verra DM, Guido ME (2006) An invertebrate-like phototransduction cascade mediates light detection in the chicken retinal ganglion cells. Faseb J 20: 2648-2650.

9. Hall MD, Hoon MA, Ryba NJ, Pottinger JD, Keen JN, et al. (1991) Molecular cloning and primary structure of squid (Loligo forbesi) rhodopsin, a phospholipase C-directed G-protein-linked receptor. Biochem J 274 ( Pt 1): 35-40.

10. Gomez MP, Nasi E (2000) Light transduction in invertebrate hyperpolarizing photoreceptors: possible involvement of a Go-regulated guanylate cyclase. J Neurosci 20: 5254-5263.

11. Kojima D, Terakita A, Ishikawa T, Tsukahara Y, Maeda A, et al. (1997) A novel Go-mediated phototransduction cascade in scallop visual cells. J Biol Chem 272: 22979-22982.

12. Maeda T, Van Hooser JP, Driessen CA, Filipek S, Janssen JJ, et al. (2003) Evaluation of the role of the retinal G protein-coupled receptor (RGR) in the vertebrate retina in vivo. J Neurochem 85: 944-956.

13. Yokoyama S, Zhang H (1997) Cloning and characterization of the pineal gland-specific opsin gene of marine lamprey (Petromyzon marinus). Gene 202: 89-93.

14. Arendt D, Tessmar K, de Campos-Baptista MI, Dorresteijn A, Wittbrodt J (2002) Development of pigment-cup eyes in the polychaete Platynereis dumerilii and evolutionary conservation of larval eyes in Bilateria. Development 129: 1143-1154.

15. Arendt D, Tessmar-Raible K, Snyman H, Dorresteijn AW, Wittbrodt J (2004) Ciliary photoreceptors with a vertebrate-type opsin in an invertebrate brain. Science 306: 869-871.

16. Engels A, Reichert H, Gehring WJ, Gartner W (2000) Functional expression of a locust visual pigment in transgenic Drosophila melanogaster. Eur J Biochem 267: 1917-1922.

17. Su CY, Luo DG, Terakita A, Shichida Y, Liao HW, et al. (2006) Parietal-eye phototransduction components and their potential evolutionary implications. Science 311: 1617-1621.
