# Supplementary material for: The Origins of Novel Protein Interactions during Animal Opsin Evolution
Source: PLoS One. 2007 Oct 17;2(10):e1054. doi: 10.1371/journal.pone.0001054 (PMC2013938; doi:10.1371/journal.pone.0001054)
Supplement: Supplementary file 2 [file pone.0001054.s003.doc]

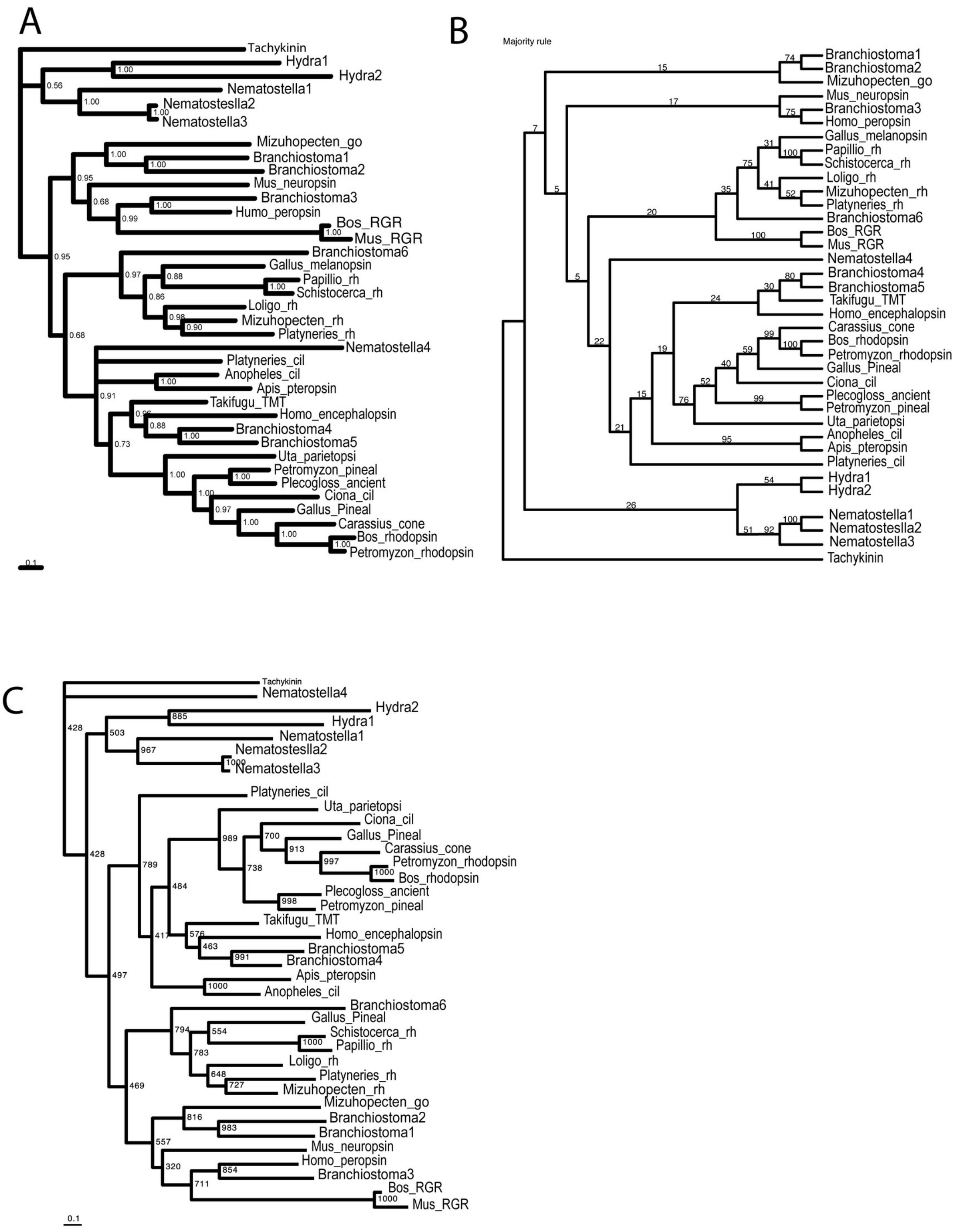
A
Tachykinin
Hydra1
Hydra2
1.00
0.56
Nematostella1
1.00
Nematostesllа2
1.00
Nematostella3
Mizuhopecten_go
1.00
Branchiostoma1
1.00
Branchiostoma2
0.95
Mus_neuropsin
Branchiostoma3
0.68
1.00
Humo_peropsin
0.99
Bos_RGR
1.00
Mus_RGR
0.95
Branchiostoma6
Gallus_melanopsin
0.97
0.88
Papillio_rh
1.00
Schistocerca_rh
0.86
Loligo_rh
0.98
Mizuhopecten_rh
0.90
Platyneries_rh
0.68
Nematostella4
Platyneries_cil
Anopheles_cil
1.00
Apis_pteropsin
0.91
Takifugu_TMT
0.96
Homo_encephalopsin
0.88
Branchiostoma4
1.00
Branchiostoma5
0.73
Uta_parietopsi
1.00
Petromyzon_pineal
1.00
Plecogloss_ancient
1.00
Ciona_cil
0.97
Gallus_Pineal
1.00
Carassius_cone
1.00
Bos_rhodopsin
1.00
Petromyzon_rhodopsin
0.1
B
Majority rule
15
74
Branchiostoma1
Branchiostoma2
Mizuhopecten_go
17
Mus_neuropsin
75
Branchiostoma3
Homo_peropsin
7
Gallus_melanopsin
31
100
Papillio_rh
Schistocerca_rh
75
Loligo_rh
5
35
41
52
Mizuhopecten_rh
Platyneries_rh
20
Branchiostoma6
100
Bos_RGR
Mus_RGR
Nematostella4
5
80
Branchiostoma4
30
Branchiostoma5
24
Takifugu_TMT
Homo_encephalopsin
99
Carassius_cone
100
Bos_rhodopsin
22
19
59
Petromyzon_rhodopsin
40
Gallus_Pineal
52
Ciona_cil
15
99
Plecogloss_ancient
76
Petromyzon_pineal
Uta_parietopsi
21
Anopheles_cil
95
Apis_pteropsin
Platyneries_cil
54
Hydra1
Hydra2
26
100
Nematostella1
51
92
Nematostesllа2
Nematostella3
Tachykinin
C
Tachykinin
Nematostella4
885
Hydra2
Hydra1
428
503
Nematostella1
967
Nematostesllа2
1000
Nematostella3
Platyneries_cil
Uta_parietopsi
Ciona_cil
428
989
700
Gallus_Pineal
913
Carassius_cone
789
997
Petromyzon_rhodopsin
738
1000
Bos_rhodopsin
484
Plecogloss_ancient
998
Petromyzon_pineal
Takifugu_TMT
417
576
Homo_encephalopsin
463
Branchiostoma5
991
Branchiostoma4
497
Apis_pteropsin
1000
Anopheles_cil
Branchiostoma6
794
Gallus_Pineal
554
Schistocerca_rh
1000
Papillio_rh
783
Loligo_rh
469
648
Platyneries_rh
727
Mizuhopecten_rh
Mizuhopecten_go
816
Branchiostoma2
983
Branchiostoma1
557
Mus_neuropsin
320
Homo_peropsin
854
Branchiostoma3
711
Bos_RGR
1000
Mus_RGR
0.1
